# Spatial and Temporal Analysis of Hospitalizations Due to Primary Care–Sensitive Conditions Related to Diabetes Mellitus in a State in the Northeast of Brazil

**DOI:** 10.3390/ijerph21111538

**Published:** 2024-11-20

**Authors:** Afonso Abreu Mendes Júnior, Álvaro Francisco Lopes de Sousa, Guilherme Reis de Santana Santos, Shirley Verônica Melo Almeida Lima, Allan Dantas dos Santos, Valdemar Silva Almeida, Ernanes Menezes dos Santos, Maria Idelcacia Nunes Oliveira, José Cleyton Santana Góis, Regina Cláudia Silva Souza, Liliane Moretti Carneiro, Maria do Carmo de Oliveira, Emerson Lucas Silva Camargo, Caíque Jordan Nunes Ribeiro

**Affiliations:** 1 Graduate Program in Nursing, Federal University of Sergipe, São Cristóvão 49107-230, SE, Brazil; afonsopadrone@gmail.com (A.A.M.J.); shirleymelo.lima@gmail.com (S.V.M.A.L.); allanufs@hotmail.com (A.D.d.S.); enffer2@gmail.com (M.d.C.d.O.); caiquejordan@academico.ufs.br (C.J.N.R.); 2 Institute of Teaching and Research, Hospital Sírio-Libanês, São Paulo 01308-050, SP, Brazil; regina.souza@hsl.org.br; 3 Postgraduate Program in Nursing, Federal University of Mato Grosso do Sul, Três Lagoas 79613-000, MS, Brazil; 4 Department of Nursing, Federal University of Sergipe, Lagarto 49400-000, SE, Brazil; guilheermereeis@gmail.com (G.R.d.S.S.); svaldemar687@gmail.com (V.S.A.); ernanessantos79@hotmail.com (E.M.d.S.); cleyton.santana020@gmail.com (J.C.S.G.); 5 Department of Nursing, Federal University of Sergipe, Aracaju 49060-025, SE, Brazil; idelnunes06@gmail.com; 6 Ribeirão Preto College of Nursing, University of São Paulo, São Paulo 14040-903, SP, Brazil; lucmrg0@gmail.com

**Keywords:** spatial analysis, diabetes mellitus, chronic disease, primary care–sensitive conditions, morbidity

## Abstract

Hospitalizations due to primary care–sensitive conditions (PCSCs) can be considered a proxy for the effectiveness of primary healthcare (PHC), especially diabetes mellitus (DM). The aim of this study was to analyze the temporal, spatial, and space–time patterns of PCSCs associated with DM in a state in Northeast Brazil from 2008 to 2022. An ecological and time–series study that included all records related to PCSCs–DM from the 75 municipalities of Sergipe was conducted. Segmented linear regression, global (I) and local (LISA) Moran indices, spatial scanning, Spearman correlation tests, bivariate I, and LISA were used in our analysis to examine the temporal trends and clusters of high spatial risk. Overall, 14,390 PCSCs–DM were recorded between 2008 and 2022. There was a higher prevalence of PCSCs–DM among women (53.75%) and individuals over 70 years old (57.60%). Temporal trends in PCSCs–DM were increasing with regard to the overall rate (AAPC: 2.39); males (AAPC: 3.15); age groups ≤ 19 years (AAPC: 6.13), 20–39 years (AAPC: 4.50), and 40–59 years (AAPC: 2.56); and 3 out of the 7 health regions. There was a positive spatial correlation between per capita income (I = −0.23; *p* = 0.004) and diabetic foot examination being performed by a nurse in a PHC (I = −0.18; *p* = 0.019) setting. The heterogeneous spatial distribution of DM hospitalizations demonstrated that this condition is a persistent public health problem in Sergipe.

## 1. Introduction

In recent decades, developing countries have experienced an accelerated epidemiological and demographic transition. This has been reflected in the growth of chronic diseases, such as diabetes mellitus (DM) [1,2], which is considered in the indicator used to measure the achievement of goal 3.4 of the United Nations Sustainable Development Goals (SDGs) [3]. This is a chronic and complex condition characterized by high glycemic levels that require multifactorial complex management. Rapid economic development and urbanization have led to an increased burden of DM in many parts of the world, and it has become a serious public health issue [4,5].

The shift to a chronic disease profile reflects the social, political, and economic transformations represented by the social determinants of health (SDH) [6], which influence the morbidity and mortality patterns of a country [7]. Low–income countries have higher rates of chronic diseases due to housing conditions, diet, and education [8]. Additionally, due to limited access to health services, fewer people with DM are diagnosed and treated in low–income countries than in high–income countries [9].

It is estimated that globally, 537 million adults (20–79 years old) live with DM, and this number is expected to increase to 643 million by 2030 and to 783 million by 2045 [10]. Its prevalence has increased in low– and middle–income countries in recent years [11], including Brazil, where in 2021 approximately 7.5 million adults (20–79 years old) lived with this disease, placing the country sixth in the world rankings [10].

The fight against this disease is a focus of major global leaders (WHO, 2016). In this context, the Global Diabetes Compact Forum was established by the WHO with the aim of strengthening the prevention, diagnosis, and treatment of DM. On average, more than 420 million people live with type 1 or type 2 DM, representing about 6% of the global population. This number is estimated to rise to over half a billion by 2030 [11].

Despite advances in DM control therapies, DM still leads to increased hospitalization and costs for the healthcare system. Therefore, it is included in the Brazilian lists of [12] hospitalizations for primary care–sensitive conditions (PCSCs) [13], which are considered powerful tools to assess the resolution, accessibility, and quality of the primary level of care. These lists refer to sets of health conditions, generally preventable, that could be effectively managed in primary healthcare (PHC) with a focus on prevention and early treatment, thereby preventing unnecessary hospitalizations [14].

Given this context, it is relevant to conduct research examining the extent of DM morbidity through spatial analysis and geoprocessing. Despite studies on PCSCs [15,16,17], few have investigated the spatial dynamics associated with DM (PCSCs–DM) through spatial, temporal, and space–time analyses.

Considering the national morbidity burden of DM and its epidemiological significance, the following research questions were proposed for this study: Was there variation in the frequency of DM–related hospital admissions (PCSCs–DM) in Sergipe from 2008 to 2022? How were PCSCs–DM distributed in this state during the period from 2008 to 2022?

As such, this study could serve as a valuable tool for public health management, guiding actions within PHC to prioritize municipalities requiring greater intervention to alter the morbidity profile and reduce secondary hospitalizations due to DM complications. Focusing on the Northeast Region of Brazil is justified because this area exhibits lower socioeconomic indicators compared to other regions of the country, including higher poverty levels, reduced access to health services, and significant social inequality. These conditions directly impact the control and management of chronic diseases, such as diabetes mellitus (DM), which require continuous and effective monitoring in primary care settings. Furthermore, the region faces disparities in terms of access to adequate healthcare in both urban and rural areas.

Therefore, our objective is to analyze the temporal, spatial, and space–time patterns of PCSCs associated with DM in a state in Northeast Brazil for the period from 2008 to 2022.

## 2. Materials and Methods

### 2.1. Study Design

This study adopts an ecological and time–series approach, utilizing publicly available data concerning PCSCs–DM. It employs the techniques of temporal, spatial, and space–time analyses, focusing on the 75 municipalities within the State of Sergipe, located in Northeast Brazil.

#### 2.1.1. Study Area

Sergipe is a state situated in the Northeast Region of Brazil, comprising 75 municipalities and divided into seven health regions (HRs). It spans a territorial area of 21,938.188 km^2^, with an estimated population of 2,338,474 individuals and a population density of 94.35 inhabitants/km^2^. The state is segmented into seven health regions (HRs), each centered around a regional headquarters: Aracaju, Estância, Lagarto, Itabaiana, Nossa Senhora do Socorro, Nossa Senhora da Glória, and Propriá.

#### 2.1.2. Study Population

The study’s population encompasses the total number of PCSCs–DM records. Causes associated with coma or ketoacidosis and complications (renal, ophthalmic, neurological, circulatory, peripheral, multiple, and others), and causes without specific complications corresponding to subcategories E.10, E.11, E.12, and E.14—as outlined in the 10th edition of the International Statistical Classification of Diseases and Related Health Problems (ICD–10)—were included. Table 1 outlines the diagnoses reported as the primary cause of hospitalization in the AIH. 

### 2.2. Data Source

The hospital morbidity data were obtained from the public database of the Hospital Information System (SIH) of the Department of Informatics of the Unified Health System (Datasus). The digital cartographic base was acquired from IBGE [18] in a shapefile format corresponding to the Universal Transverse Mercator (UTM) system and the latitude/longitude geographic projection model (Geodesic Reference System—SIRGAS 2000) of the State of Sergipe.

#### 2.2.1. Variables and Measures

The primary outcome of this study was the percentage of PCSCs–DM in Sergipe. This was calculated by dividing the number of hospitalizations due to DM by the total number of PCSCs, excluding delivery–related hospitalizations (ICD–10: O80–O84) from the denominator, and multiplying by 100.

The socioeconomic, demographic, and healthcare assistance factors analyzed in this study as independent variables were resident population, age group, race/ethnicity, gender, place of residence, health region, year of service, service type, regime, per capita income, Human Development Index (HDI), Social Vulnerability Index (SVI), primary healthcare coverage, performance indicator 7 of the *Previne Brasil* Program, the proportion of nurse and physician consultations with DM being the managed condition, and the proportion of diabetic foot examinations performed by nurses and physicians.

#### 2.2.2. Data Analysis

A descriptive analysis of the healthcare and epidemiological profile and demographic factors related to hospitalizations due to DM in the State of Sergipe was conducted. Temporal trends were analyzed using joinpoint regression models (segmented linear regression). For trend calculation, PCSCs–DM rates (by gender and age group) were considered dependent variables, and the year of an event’s occurrence was the independent variable. This method allows for the assessment of changes in the trend of the indicator over time by fitting data from a series using the fewest possible joinpoints and examining if the inclusion of more joinpoints is significant.

The Monte Carlo permutation test was used to select the best segment for each model. The best–fitting model was the one with the highest coefficient of determination of residuals (R2). Subsequently, the annual percent change (APC) and its respective 95% confidence interval (95% CI) were calculated for each segment to describe and quantify the trend and assess its statistical significance [19].

The average annual percent change (AAPC) for the entire period was calculated to simplify the comparison of trends for indicators with more than one significant slope in the period. An estimate was obtained using the weighted geometric mean of the APC, with weights equal to the length of each time interval of the segment. Trends were statistically significant when APCs and AAPCs had *p*–values < 0.05 and when their 95% CIs did not include zero.

To investigate the spatial autocorrelation of PCSCs–DM rates, the global Moran’s index (I) was calculated, ranging from −1 to +1, where positive values indicate direct correlation, negative values denote inverse correlation, and zero indicates a lack of autocorrelation [20].

Once spatial dependence was identified through I, the occurrence of local autocorrelation was assessed by calculating the univariate Local Indicators of Spatial Association (LISA) and Moran’s Index. This index determined the data dependence among neighboring regions, enabling the identification of spatial association patterns that may indicate the occurrence of spatial clusters of municipalities [20]. The space–time scan analysis was conducted to identify high–risk clusters for PCSCs–DM. This analysis examines spatial dependence and risk patterns for the disease by representing quadrants: Q1 (high/high) and Q2 (low/low) indicate municipalities with similar values to their neighbors, i.e., areas of concordance, while Q3 (high/low) and Q4 (low/high) have different values, indicating transition areas with negative spatial associations [20].

The correlation between PCSCs–DM rates, SDH, and healthcare indicators was calculated using Spearman’s correlation (Rho). Thematic maps of the indicators showing significant correlation with PCSCs–DM rates in Sergipe were created. Univariate I and LISA calculations were performed to examine the spatial autocorrelation of the indicators significantly associated with PCSCs–DM. The scatterplot results for each indicator were represented by Moran maps for visual comparison. Subsequently, bivariate I and LISA calculations were performed to verify the spatial correlation between PCSCs–DM, SDH, and the healthcare indicators of the study municipalities. The results of this stage were also plotted on Moran maps. Only results with *p*–values < 0.05 were visually represented.

#### 2.2.3. Software

The data were stored in a Microsoft Office Excel 2016 spreadsheet (Microsoft Corporation; Redmond, WA, USA). All data analyses, thematic maps, spatial analyses, and temporal trend analyses were conducted using R software, version 4.3.1 (The R Core Team, 2022).

## 3. Results

### 3.1. Demographic Data

A total of 198,781 PCSCs were recorded in Sergipe from 2008 to 2022, of which 14,390 were due to DM (7.24%). Most hospitalizations occurred among women (n = 7734; 53.75%), people of mixed race (n = 3690; 87.30%), and people aged ≥ 60 years (n = 8289; 57.60%) (Table 2).

#### 3.1.1. Temporal Trend Analysis

**Figure 1:**
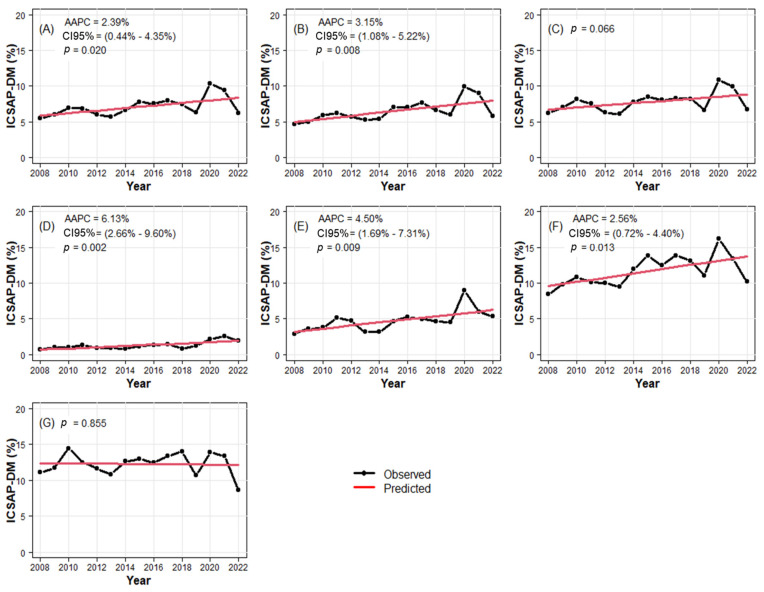
Temporal trends in PCSCs–DM by state, gender, and age group for Sergipe, Northeast, Brazil, 2008–2022. (**A**): Entire state. (**B**): Male. (**C**): Female. (**D**): Age group: <19 years. (**E**): Age group: 20 to 39 years. (**F**): Age group: 40 to 59 years. (**G**): Age group: ≥60 years.

The analysis of temporal trends was conducted using segmented linear regression. Figure 1A depicts the trend in the rate of PCSCs–DM in Sergipe over the years, showing a significant increase (*p* < 0.05) with an average annual percentage change (AAPC) of 2.39% (95% CI: 0.44–4.35). The trends in PCSCs–DM rates were found to be increasing among males (AAPC: 3.15%; *p* = 0.008; 95% CI: 1.08–5.22) (Figure 1B) and age groups of 0–19 years (AAPC: 6.13%; *p* = 0.002; 95% CI: 2.66–9.60) (Figure 1D), 20–39 years (AAPC: 4.50%; *p* = 0.009; 95% CI = 1.69–7.31) (Figure 1E), and 40–59 years (AAPC: 2.56%; *p* = 0.013; 95% CI: 0.72–4.40) (Figure 1F). Despite the faster increase in the 0–19 age group, percentage changes remained lower (<5%). Meanwhile, the temporal trend in those aged over 60 years was considered stable, with rates ranging between 10% and 15% (Figure 1G) (Table 3).

#### 3.1.2. Spatial and Space–Time Cluster Analysis

**Figure 2:**
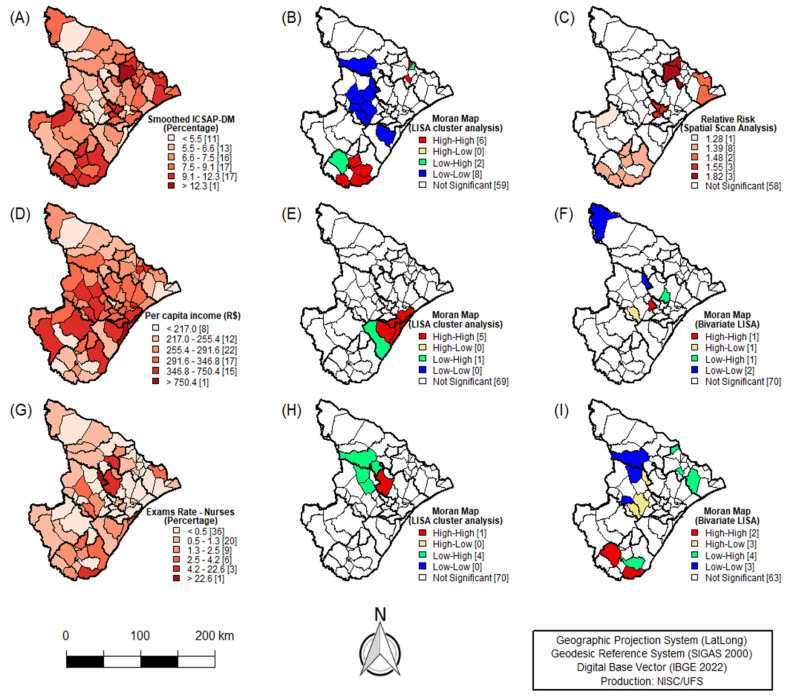
Spatial distribution of PCSCs–DM. Sergipe, Northeast, Brazil, 2008–2022. (**A**): Smoothed PCSCs–DM (%). (**B**): Moran map (Univariate LISA). (**C**): Relative risk (spatial scan analysis). (**D**): Per capita income (R$). (**E**): Moran map per capita income (univariate LISA). (**F**): Moran map per capita income (bivariate LISA). (**G**): Nurse examination rate (%). (**H**): Moran map nurse examination rate (univariate LISA). (**I**): Moran map nurse examination rate (bivariate LISA).

Figure 2A represents the distribution of smoothed rates found using the Empirical Bayesian Local method of PCSCs–DM assessment in the territory of Sergipe. In most municipalities (51/75; 68%), DM accounted for more than 6% of cases of PCSCs. Additionally, this distribution did not occur randomly, as evidenced by global spatial autocorrelation (I = 0.32; *p* = 0.001). Univariate LISA analysis identified spatial clusters of municipalities with similar spatial patterns, highlighting the high–risk cluster encompassing six municipalities, two from the Propriá health region (Amparo de São Francisco and Malhada dos Bois), and four from the Estância health region (Arauá, Cristinápolis, Indiaroba, and Santa Luzia do Itanhy) (Figure 2B).

Spatial scan analysis identified five statistically significant clusters of PCSCs–DM, as visualized in Figure 2C. Clusters with higher relative risk (RR = 1.82; *p* < 0.001) were detected, including three municipalities of the Propriá health region (Aquidabã, Canhoba, and Muribeca). This applied to a lesser extent (RR = 1.28; *p* < 0.001) to the municipality of the Lagarto health region (Simão Dias) (Figure 2C). The clustering pattern observed supports the findings regarding the spatial distribution of smoothed rates and univariate LISA analysis (Table 4).

To identify the variables associated with PCSCs–DM in Sergipe, bivariate analyses were performed. Initially, Spearman’s linear correlation test indicated a significant association between PCSCs–DM rates, per capita income (Rho = −0.23; *p* = 0.004), and diabetic foot examinations performed by nurses (Rho = −0.18; *p* = 0.019). The spatial distribution of these independent variables revealed that the majority of Sergipe municipalities have incomes ≤ R$291.60 (42/75; 56%) and represent a percentage ≤ 1.3% of diabetic foot examinations performed by nurses in primary care (56/75; 74.6%) (Figure 2D,G) (Table 5).

Subsequently, univariate Moran analysis of the aforementioned variables revealed the spatial autocorrelation for per capita income (I = 0.25; *p* = 0.004) and diabetic foot examinations performed by nurses (I = 0.03; *p* = 0.019), indicating that municipalities in the Aracaju health region (Aracaju, Barra dos Coqueiros, and São Cristóvão) and the Nossa Senhora do Socorro region (Nossa Senhora do Socorro and Santo Amaro das Brotas) showed clustering with regard to higher per capita income (Figure 2E). Meanwhile, the municipality of Nossa Senhora das Dores, belonging to the Nossa Senhora do Socorro health region, was the only one that presented a cluster of more diabetic foot examinations performed by nurses (Figure 2H) (Table 5).

The Campo do Brito municipality in the Itabaiana health region showed a high rate of PCSCS–DM and comprised the transition cluster, being surrounded by neighbors with low per capita income levels (Figure 2F). Bivariate LISA analysis demonstrated that municipalities with lower hospitalization rates have a higher rate of diabetic foot examinations performed by nurses in primary care, with São Miguel do Aleixo municipality (Q3: High–Low) in the Itabaiana region standing out. Here, 37.55% of diabetic foot examinations were performed by nurses and there was a hospitalization rate of 7.10%, as shown in Figure 2I.

## 4. Discussion

This study marks a significant advancement in terms of performing spatial and temporal analysis of PCSCs–DM in the State of Sergipe, spanning a comprehensive period from 2008 to 2022. The innovative aspect of this research lies in its comprehensive and multidimensional approach, integrating time–series analyses, spatial analysis techniques, and spatial scan statistics to map the trends and patterns of PCSCs–DM. This study not only revealed the escalating prevalence of DM to be a leading cause of hospitalizations but also highlighted significant variations in PCSCs–DM rates across different age groups, genders, and health regions within the state.

The findings of this study underscore the critical importance of interventions in primary healthcare (PHC), emphasizing the need for policymakers to strengthen care for patients with chronic conditions and allocate resources to promote the prevention of non–communicable diseases (NCDs) in priority areas. This aligns with SDG target 3.4, which necessitates globally coordinated efforts [3]. While our study indicates the high prevalence of PCSCs–DM in the elderly population [21,22], akin to findings attributing the global rise in DM to population aging, we observed a stable trend in hospitalizations among this age group.

This disparity may stem from various factors. Primarily, disparities in healthcare policies and the implementation of PHC across different states can influence the efficacy of DM management and, consequently, hospitalization rates. Although the higher prevalence of PCSCs among the population aged over 70 years in the North and Northeast Regions has prompted the strengthening of public policies for the elderly [23], our study suggests a stable trend in hospitalizations within this age group. This might reflect specificities in the care and health management of the elderly in Sergipe, indicating that local PHC strategies may effectively mitigate hospitalizations in this demographic.

Furthermore, it is imperative to consider clinical heterogeneity and the co–occurrence of comorbidities among the elderly. The unique nature of DM management in this demographic necessitates a comprehensive, interdisciplinary, and multidimensional approach. While DM itself is a risk factor for hospitalizations, the effective management of associated comorbidities and the implementation of health promotion and prevention strategies may contribute to the stability observed in our findings.

Despite the global and national trend of increased PCSCs–DM with advancing age, data from Sergipe show a distinct pattern. This peculiarity suggests the possible influence of factors such as the effectiveness of local health policies, DM management practices in PHC, and the specific sociodemographic and health characteristics of the elderly population in the state. Another important finding of this study is the emerging growth of PCSCs–DM in the age group < 19 years, although this trend is in its early stages. This outcome underscores the importance of consolidating programs and policies focused on child and adolescent health, such as the Health in Schools Program (HSP), with an emphasis on promoting healthy eating habits and growth. Indeed, a study in Michigan, USA, demonstrated the significance of school interventions in DM prevention, reaffirming the pivotal role of the HSP as a strategic tool in PHC with which to foster health in the school setting [24].

In terms of gender, our study observed that women were predominantly affected, in line with a previous study [25]. However, this trend seems to diverge from a study indicating the growing prevalence of DM among men [26]. The increasing trend of hospitalizations among men suggests the need for healthcare policies that are more focused on the male population, considering that, traditionally, women tend to take better care of their health and be more attentive to weight control [27].

It is important to note that, in addition to common risk factors for both genders, women face additional risks such as gestational diabetes (GDM) and polycystic ovary syndrome [4]. These specific factors may contribute to the higher frequency of hospitalizations among women observed in our study.

Therefore, our results indicate that there are important nuances in the profile of DM hospitalizations that should be considered in the development of health policies and intervention strategies. While the emphasis on child and adolescent health is supported by the increase in PCSCs–DM among individuals under 19 years old, the growing trend of hospitalizations among men points to the need for more inclusive healthcare approaches attentive to gender–specific particularities. Additionally, the greater vulnerability of women, given the presence of specific risk factors, reinforces the need for public health strategies that comprehensively and contextually address the challenges posed by DM.

Interestingly, a study conducted in Tunisia, Africa, revealed increasing trends in the occurrence of diabetic ketoacidosis through segmented linear regression analysis (joinpoint), which was also utilized to examine temporal trends in our study. The authors further emphasize that this trend was even more significant during the COVID–19 pandemic, suggesting that there may have been delayed diagnoses or, through an autoimmune trigger mechanism, an inflammatory environment conducive to insulin resistance [28].

A study conducted in the United States showed that there is an increased risk of hospital readmission among black diabetic patients compared to white patients. Although the reason for readmission is multifactorial, the increased risk is more evident among low–income patients [29]. The present study complements the existing literature, suggesting that non–white populations may have a higher number of PCSCs–DM patients.

Thus, reducing health disparities among ethnic and racial minorities provides equitable DM care in line with one of the principles of SUS. Therefore, given that the management and complications of DM differ among racial and ethnic subgroups [30], developing more equitable health policies and strategies to address DM targeted at subgroups will make healthcare systems more effective, as SDHs are fundamental in healthcare provision.

Considering that DM emerged as one of the most concerning and dangerous NCDs, reaching pandemic proportions with a global prevalence of 9% in 2019 [21], when analyzing hospitalization rates for causes associated with the condition in Sergipe, a significant increase was observed over the years in the state, similarly to what was observed in Brazil, where the absolute number of hospitalizations due to this condition increased by 1.83% [25]. Without effective and urgent interventions, it is predicted that by 2045, 700 million people will have DM [31]; consequently, hospitalizations and deaths due to this condition will show a significant increase. Therefore, efforts and commitments are necessary in all health regions of the State of Sergipe to reduce PCSCs–DM and contribute to the achievement of SDG target 3.4.

Although the Aracaju health region has the highest per capita income, a high rate of PCSCs–DM [26] was observed, a paradoxical phenomenon, since high–income countries have a lower prevalence of DM. Furthermore, local studies have shown a downward trend in hospitalizations for DM, contrasting with the findings in Aracaju [32,33].

This seemingly contradictory scenario in Aracaju can be elucidated by considering the complexities introduced by globalization and unplanned urbanization, which generate significant social inequalities within the same country [34]. The reality of Aracaju, therefore, may reflect the existence of hidden ‘pockets of poverty’ where significant segments of the population live in conditions of vulnerability, despite apparent overall prosperity. This phenomenon was previously observed in a study that identified a positive correlation between urbanization, population density, and an increase in tuberculosis in Sergipe [35].

Thus, the need is evident for healthcare policies that go beyond the analysis of general economic indicators and address the intrinsic socio–spatial disparities within metropolitan regions. Identifying and directing resources and interventions to the most distant and deprived neighborhoods, where the most vulnerable populations reside, is essential to mitigate health disparities.

Economic determinants such as per capita income can influence the population’s access to various behaviors that impact health conditions, such as engaging in physical activity and maintaining a healthy diet [34]. This fact is in line with the results of this study, as the higher per capita income in municipalities in the State of Sergipe was associated with a lower number of hospitalizations due to DM. Therefore, DM needs to be approached with a multidimensional vision, considering socioeconomic and environmental factors. This requires the adoption of a shared approach by professionals, users, and the community, focusing on preventing risk factors and understanding multiple individual needs.

Another noteworthy result in this study concerns the attention to diabetic foot care in primary care settings, as our findings showed that, overall, diabetic foot examination is insufficient, despite diabetic foot ulcers being associated with high rates of morbidity and mortality. Examination by a professional should be performed annually [36]. Although primary prevention is fundamental, tertiary prevention can result in a reduction in the level of PCSCs–DM.

A study conducted in Brazil showed that weaknesses in diabetic care in primary care result in worse health conditions and higher complications and hospitalizations due to DM [37]. A systematic review highlights the importance of developing interventions, according to patient conditions and needs, to prevent foot ulcers in patients with DM [38]. Therefore, primary care is the ideal level of care for managing DM and has the capacity to substantially reduce PCSCs–DM and bring about the early prevention of complications.

In Sergipe, the performance of diabetic foot examination by nurses occurred at a proportionally higher rate than that performed by physicians, reinforcing the nurses’ care perspective during the consultation in terms of conducting the diabetic foot examination and offering self–care guidance as it is a preventable condition that can cause amputations and unnecessary hospitalizations [39,40]. The results showed that municipalities with a higher rate of diabetic foot examination performed by primary care nurses had a lower rate of PCSCs–DM, confirming the need for the implementation of robust care pathways in primary care in the care of people with DM according to the protocols of the Ministry of Health.

The present study demonstrates to primary care professionals the importance of understanding the relationship between glycemic control and morbidity and mortality due to DM, emphasizing the need to achieve glycemic targets through HbA1c control. In this context, the study’s results emphasize the relevance of primary care professionals requesting HbA1c tests, which contribute to the achievement of indicator 7 of the *Previne Brasil* Program. This indicator aims to identify contact between individuals with DM and healthcare services, enabling care provision, HbA1c testing for glycemic level assessment, the determination of condition control, and the prevention of morbidity and mortality.

Collectively, the findings of this study demonstrate a comprehensive understanding of a reality common to other states and nations, indicating the urgent need to organize healthcare services, particularly in primary care. Strategies and interventions aimed at meeting the health needs of the population are necessary to create a healthcare system that is proactive in addressing chronic diseases and achieving SDG targets.

Initially, policymakers and managers must recognize the necessity of incorporating a care model focused on chronic diseases while strengthening primary care. This level of care is pivotal in managing NCDs and preventing hospitalizations, particularly those related to PCSCs–DM. Moreover, recognizing the significance of the nurse’s role in DM care can reduce hospitalizations and complications, effectively responding to shifts in the clinical and epidemiological landscape.

In the context of limited resources or vulnerable areas, understanding DM and its determining factors becomes crucial. Barriers to accessing diagnosis, treatment, and adequate medical care for the disease are amplified in these locations, precisely where vulnerable populations face obstacles such as a lack of financial resources, a precarious medical infrastructure, and a shortage of qualified health professionals. This combination of factors further aggravates the problem. Therefore, DM management should prioritize the development of therapeutic plans that consider the choices and life context of individuals living with DM. This includes educational initiatives, simplified language, therapeutic groups, and the integration of multiprofessional teams to support individuals with DM in taking charge of their self–care and managing their health situation more effectively. Therefore, the use of lightweight technologies contributes to providing care for patients with DM and should be prioritized in primary care settings facing vulnerabilities.

Understanding the spatial behavior of DM is crucial for equitable resource allocation and effective interventions, especially in a country with vast geographic and socioeconomic diversity. Given our findings, analyzing DM hospitalizations as a public health burden necessitates healthcare attention being focused on chronic diseases and the consolidation of promotion and prevention efforts. DM remains a challenge, requiring mutual and collective efforts from users, professionals, and managers to ensure access to healthcare resources.

Our study underscores the importance of using spatial and temporal tools to map vulnerable areas requiring more attention. Spatial and temporal analyses are indispensable in comprehending the dynamics of diseases and conditions within a territory when aiming to identify areas with increasing trends, which necessitate prioritization and targeted interventions, as well as transition areas requiring promotion and prevention measures.

There is an urgent need to develop equitable public policies to improve living and health conditions for populations residing in impoverished areas and municipalities with low levels of economic development. Undoubtedly, the results highlight the strong correlation between social and economic disparities and the heightened distribution of diseases and conditions, indicating the necessity for representative, resilient, and sustainable social policies.

Despite its strengths, the results of this study are not without limitations, as underreporting, incorrect records, and incomplete or low–quality data may affect the accuracy of frequency distributions. Additionally, the COVID–19 pandemic may have impacted data reporting due to the strain on healthcare systems and the physical and mental exhaustion of professionals, making them prone to errors and technical failures. The increased demand for health services and the reorganization of care to prioritize COVID–19 cases may have affected the monitoring and management of diabetes, leading to changes in hospitalization rates due to DM complications. This situation may also have contributed to the rise in avoidable hospitalizations during the final period of analysis. Moreover, future studies focused on the direct and indirect impacts of the pandemic on chronic disease management may offer a more detailed understanding of this phenomenon.

Furthermore, the choice of spatial units (municipalities) can influence the detection of autocorrelation. Aggregating data into larger spatial units may obscure existing autocorrelation patterns at more localized levels; for example, a municipal–level analysis may fail to detect disease clusters present in specific neighborhoods within municipalities. The strength and type of autocorrelation may even vary within the study area itself. While the Moran’s Index and LISA provide an overview, they may not capture local nuances effectively.

## 5. Conclusions

In conclusion, PCSCs–DM in Sergipe showed an upward trend, which was particularly notable in 2017. These issues were more prevalent among women, individuals of non–white ethnicity, and those aged 60 years or older. The nature of the care provided was predominantly urgent, and the type of healthcare facility involved was primarily private. Increasing trends in hospitalizations for DM were also noted among males in the age groups of <19 years, 20–39 years, and 40–59 years.

PCSCs–DM are not randomly distributed across the territory of Sergipe, exhibiting spatial dependence regarding their occurrence in the health regions of Estância, Propriá, and Itabaiana, particularly in the municipalities of Aquidabã (Propriá region), Riachuelo (Aracaju region), and Umbaúba (Estância region), where there is a clustering of high spatial and spatiotemporal risk.

The per capita income of municipalities and diabetic foot examinations conducted by nurses showed the significant spatial autocorrelation of high–risk clusters for DM hospitalizations. Bivariate analysis indicated a higher rate of occurrence of PCSCSs–DM in municipalities with lower incomes, especially in Siriri (Nossa Senhora do Socorro region), and in municipalities with higher rates of PCSCs–DM and fewer diabetic foot examinations conducted by nurses, particularly Japoatã, Malhada dos Bois, and Nossa Senhora de Lourdes (Propriá region), as well as Santa Luzia do Itanhy and Tomar do Geru (Estância region).

Our findings highlight the urgent need for a response involving effective interventions in prioritized municipalities. Such a response should primarily focus on providing equitable attention to areas with racial and socioeconomic disparities, as Brazil is a country marked by social health inequities, and social determinants of health (SDH) can significantly affect DM–related trends and exert a strong influence on the prevalence of DM and its complications.

## Figures and Tables

**Table 1:** Primary diagnoses reported as the cause of hospitalization, as per the morbidity tabulation list. Sergipe, SE, Brazil, 2023.

Group	Diagnosis of DM	ICD–10 Code
(14.0 Diabetes)	With coma or ketoacidosis	E10.0–E10.1, E11.0–E11.1, E12.0–E12.1, E13.0–E13.1, E14.0–E14.1
14.1	Without specific complications	E10.9, E11.9 E12.9, E13.9 E14.9
14.2	With complications (renal, ophthalmic, neurological, circulatory, peripheral, other, multiple, unspecified)	E10.2–E10.8, E11.2–E11.8, E12.2–E12.8, E13.2–E13.8, E14.2–E14.8

**Table 2:** Baseline characteristics of the study population. Sergipe, Northeast, Brazil, 2008–2022.

Variable	n	%
Gender		
Male	6656	46.25
Female	7734	53.75
Skin color/race		
Black	99	2.34
Yellow	180	4.26
White	256	6.06
Brown	3.690	87.30
Age group		
<19 years	964	6.70
20 to 39 years	1.049	7.29
40 to 59 years	4088	28.41
60+ years	8289	57.60
Type of health service delivery system		
Public	2066	31.47
Private	4498	68.53
Nature of care		
Elective	320	2.22
Urgency	14,070	97.78

**Table 3:** Temporal trends in analyzed variables. Sergipe, Northeast Brazil, 2008–2022.

Variables	Period	AAPC (IC95%)	*p*–Value	Trend
PCSCs–DM rate	2008–2022	2.39% (0.44 to 4.35)	0.020	Increasing
Gender				
Male	2008–2022	3.15% (1.08 to 5.22)	0.008	Increasing
Female	2008–2022	1.63% (−0.55 to 3.81)	0.066	Stable
Age group				
<19 years	2008–2022	6.13% (2.66 to 9.60)	0.002	Increasing
20 to 39 years	2008–2022	4.50% (1.69 to 7.31)	0.009	Increasing
40 to 59 years	2008–2022	2.56% (0.72 to 4.40)	0.013	Increasing
≥60 years	2008–2022	−0.58% (−2.49 to 1.32)	0.085	Stable
Health Regions				
Aracaju	2008–2022	2.92% (0.68 to 5.17)	0.017	Increasing
Estância	2008–2022	4.31% (0.97 to 7.64)	0.021	Increasing
Propriá	2008–2022	7.45% (5.27 to 9.64)	0.019	Increasing

**Table 4:** Space–time clusters of PCSCs–DM. Sergipe, Northeast Brazil, 2008–2022.

Cluster	Number of Municipalities	Health Regions	Number of Cases	RR	Statistical Value	*p*–Value
1	3	Propriá	1409	1.82	222.86	0.001
2	8	Estância	1864	1.39	101.09	0.001
3	3	Aracaju	959	1.55	83.31	0.001
4	2	Propriá	227	1.48	19.13	0.001
5	1	Lagarto	266	1.28	7.63	0.044

**Table 5:** Spatial autocorrelation of PCSCs–DM and its association with per capita income and diabetic foot exams performed by nurses from primary healthcare teams. Sergipe, Northeast Brazil, 2008–2022.

Variable	Correlation Rho	*p*–Value	Univariate Moran’s I	*p*–Value	Bivariate Moran’s I	*p*–Value
Performance indicator	0.05	0.546	0.00	0.932	0.23	0.978
Per capita income	−0.23	0.004	0.25	0.001	−0.29	0.006
HDM	−0.07	0.344	0.55	0.000	−0.10	0.203
Gini coefficient	−0.11	0.160	0.22	0.025	−0.26	0.011
SVI	0.09	0.258	0.38	0.000	0.40	1.000
Diabetic foot examination (Performed by nurse)	−0.18	0.019	0.03	0.838	−0.52	0.001
PHC consultations (Performed by nurse)	−0.02	0.787	0.18	0.065	0.22	0.971
Diabetic Foot Examination (Performed by doctor)	−0.15	0.102	−0.04	0.737	0.15	0.903
PHC consultations (Performed by doctor)	−0.09	0.262	0.32	0.001	0.23	0.977
Estimated population coverage by PHC teams	0.03	0.722	0.17	0.014	0.14	0.876

## Data Availability

All data used and analyzed are available in the public domain. Morbidity data were provided by Datasus through the Hospital Information Systems databases, Available on https://datasus.saude.gov.br/informacoes-de-saude-tabnet/ (accessed on 17 November 2024). Socioeconomic and demographic data were collected through the databases of the 2010 Census of the Brazilian Institute of Geography and Statistics and the Institute of Applied Economic Research (IPEA).

## References

[1] Calazans J.A., Queiroz B.L. (2020). The adult mortality profile by cause of death in 10 Latin American countries (2000–2016). Rev. Panam. Salud Publica.

[2] Cousin E., Schmidt M.I., Ong K.L., Lozano R., Afshin A., Abushouk A.I., Agarwal G., Agudelo–Botero M., Al–Aly Z., Alcalde–Rabanal J.E. (2022). Burden of diabetes and hyperglycaemia in adults in the Americas, 1990–2019: A systematic analysis for the Global Burden of Disease Study 2019. Lancet Diabetes Endocrinol..

[3] Bennett J.E., Kontis V., Mathers C.D., Guillot M., Rehm J., Chalkidou K., Kengne A.P., Carrillo–Larco R.M., Bawah A.A., Dain K. (2020). NCD Countdown 2030: Pathways to achieving Sustainable Development Goal target 3.4. Lancet.

[4] ElSayed N.A., Aleppo G., Aroda V.R., Bannuru R.R., Brown F.M., Bruemmer D., Collins B.S., Gaglia J.L., Hilliard M.E., Isaacs D. (2023). 2. Classification and Diagnosis of Diabetes: Standards of Care in Diabetes—2023. Diabetes Care.

[5] Abdul Basith Khan M., Hashim M.J., King J.K., Govender R.D., Mustafa H., Al Kaabi J. (2019). Epidemiology of Type 2 Diabetes—Global Burden of Disease and Forecasted Trends. J. Epidemiol. Glob. Health.

[6] Hatefi A., Allen L.N., Bollyky T.J., Roache S.A., Nugent R. (2018). Global susceptibility and response to noncommunicable diseases. Bull. World Health Organ..

[7] Stringhini S., Carmeli C., Jokela M., Avendaño M., Muennig P., Guida F., Ricceri F., d’Errico A., Barros H., Bochud M. (2017). Socioeconomic status and the 25 × 25 risk factors as determinants of premature mortality: A multicohort study and meta–analysis of 1·7 million men and women. Lancet.

[8] Ezzati M., Pearson-Stuttard J., Bennett J.E., Mathers C.D (2018). Acting on non–communicable diseases in low–and middle–income tropical countries. Nature.

[9] Di Cesare M., Khang Y.-H., Asaria P., Blakely T., Cowan M.J., Farzadfar F., Guerrero R., Ikeda N., Kyobutungi C., Msyamboza K.P. (2013). Inequalities in non–communicable diseases and effective responses. Lancet.

[10] International Diabetes Federation (2021). IDF Diabetes Atlas 2021|IDF Diabetes Atlas.

[11] World Health Organization (2020). Global Health Estimates: Leading Causes of Death. https://www.who.int/data/gho/data/themes/mortality-and-global-health-estimates/ghe-leading-causes-of-death.

[12] Harding J.L., Pavkov M.E., Magliano D.J., Shaw J.E., Gregg E.W. (2019). Global trends in diabetes complications: A review of current evidence. Diabetologia.

[13] Brasil Portaria no. 221, de 17 de Abril de 2008. https://bvsms.saude.gov.br/bvs/saudelegis/sas/2008/prt0221_17_04_2008.html.

[14] Alfradique M.E., Bonolo P.D.F., Dourado I., Lima–Costa M.F., Macinko J., Mendonça C.S., Oliveira V.B., Sampaio L.F.R., Simoni C.D., Turci M.A. (2009). Internações por condições sensíveis à atenção primária: A construção da lista brasileira como ferramenta para medir o desempenho do sistema de saúde (Projeto ICSAPs—Brasil). Cad. Saude Publica.

[15] Freitas J.L.G., Silva P.P.D., Moreira K.F.A., Cavalcante D.F.B., Souza M.H.D.N., Alves J.C. (2020). Internações por condições sensíveis à atenção primária em crianças em Rondônia de 2008 a 2017. Cogitare Enferm..

[16] Pinto Junior E.P., Aquino R., Dourado I., Costa L.D.Q., Silva M.G.C.D. (2020). Internações por condições sensíveis à Atenção Primária à Saúde em crianças menores de 1 ano no Brasil. Cien. Saude Colet..

[17] Chen S., Fu H., Jian W. (2022). Trends in avoidable hospitalizations in a developed city in eastern China: 2015 to 2018. BMC Health Serv. Res..

[18] IBGE Instituto Brasileiro de Geografia E Estatísticas. https://cidades.ibge.gov.br/.

[19] Antunes J.L.F., Cardoso M.R.A. (2015). Uso da análise de séries temporais em estudos epidemiológicos. Epidemiol. E Serviços Saúde.

[20] Anselin L. (1995). Local Indicators of Spatial Association—LISA. Geogr. Anal..

[21] Sun H., Saeedi P., Karuranga S., Pinkepank M., Ogurtsova K., Duncan B.B., Stein C., Basit A., Chan J.C., Mbanya J.C. (2022). IDF Diabetes Atlas: Global, regional and country–level diabetes prevalence estimates for 2021 and projections for 2045. Diabetes Res. Clin. Pract..

[22] Viacava F., Carvalho C., Martins M., Oliveira R. (2022). Internações por Condições Sensíveis à Atenção Primária (ICSAPs): Análise descritiva por sexo e idade e diagnósticos principais. Boletim Informativo do PROADESS, no. 9, out./ 2022.

[23] Moura F., Salles J.E.N., Valente F., Almeida–Pititto B.D., Fonseca R.M.C., Cavalcanti S., Bertoluci M. (2023). Abordagem do paciente idoso com diabetes mellitus. Diretriz da Sociedade Brasileira de Diabetes.

[24] Nurjannah N., Baker K.M. (2020). Using Gis and Death Records to Inform Statewide School–Based Diabetes Prevention Interventions in Michigan. J. Public Health Res..

[25] Florêncio R.B., Fonseca L.G.d.A., da Silva V.F.D., Lima Í.N.D.F., Gualdi L.P. (2021). Diabetes mellitus hospitalization and mortality rate according to a national database in Brazil: A longitudinal study. BMC Public Health.

[26] Lovic D., Piperidou A., Zografou I., Grassos H., Pittaras A., Manolis A. (2020). The Growing Epidemic of Diabetes Mellitus. Curr. Vasc. Pharmacol..

[27] Harreiter J., Kautzky–Willer A. (2018). Sex and Gender Differences in Prevention of Type 2 Diabetes. Front. Endocrinol..

[28] Gorchane A., Ach T., Sahli J., Abdelkrim A.B., Mallouli M., Bellazreg F., Hachfi W., Chaieb M.C., Ach K. (2023). Uncovering the alarming rise of diabetic ketoacidosis during COVID–19 pandemic: A pioneer African study and review of literature. Front. Endocrinol..

[29] Rodriguez–Gutierrez R., Herrin J., Lipska K.J., Montori V.M., Shah N.D., McCoy R.G. (2019). Racial and Ethnic Differences in 30-Day Hospital Readmissions Among US Adults with Diabetes. JAMA Netw. Open.

[30] Hassan S., Gujral U.P., Quarells R.C., Rhodes E.C., Shah M.K., Obi J., Lee W.-H., Shamambo L., Weber M.B., Narayan K.M.V. (2023). Disparities in diabetes prevalence and management by race and ethnicity in the USA: Defining a path forward. Lancet Diabetes Endocrinol..

[31] Saeedi P., Petersohn I., Salpea P., Malanda B., Karuranga S., Unwin N., Colagiuri S., Guariguata L., Motala A.A., Ogurtsova K. (2019). Global and regional diabetes prevalence estimates for 2019 and projections for 2030 and 2045: Results from the International Diabetes Federation Diabetes Atlas, 9th edition. Diabetes Res. Clin. Pract..

[32] Santos A.L., Teston E.F., Latorre M.R.D.O., Mathias T.A.F., Marcon S.S. (2015). Tendência de hospitalizações por diabetes mellitus: Implicações para o cuidado em saúde. Acta Paul. Enferm..

[33] Souza Júnior E.V., Jesus M.A.S., Lapa P.S., Cruz J.S., Maia T.F., Barros V.S., Almeida N.S., Boery E.N. (2019). Internações, óbitos e custos hospitalares por diabetes mellitus. Rev. Enferm. UFPE Online.

[34] Carrapato P., Correia P., Garcia B. (2017). Determinante da saúde no Brasil: A procura da equidade na saúde. Saúde E Soc..

[35] Lima S.V.M.A., Dos Santos A.D., Duque A.M., de Oliveira Goes M.A., da Silva Peixoto M.V., da Conceição Araújo D., Ribeiro C.J.N., Santos M.B., de Araújo K.C.G.M., Nunes M.A.P. (2019). Spatial and temporal analysis of tuberculosis in an area of social inequality in Northeast Brazil. BMC Public Health.

[36] Sacco I.C., Lucovéis M.D.L.S., Suely R.T., Parisi M.C.R. (2023). Diagnóstico e prevenção de úlceras no pé diabético. Diretriz da Sociedade Brasileira de Diabetes.

[37] Muzy J., Campos M.R., Emmerick I., Silva R.S., Schramm J.M.A. (2021). Prevalência de diabetes mellitus e suas complicações e caracterização das lacunas na atenção à saúde a partir da triangulação de pesquisas. Cad. Saude Publica.

[38] Rismayanti I.D.A., Nursalam N., Farida V.N., Dewi N.W.S., Utami R., Aris A., Agustini N.L.P.I.B. (2022). Early Detection to Prevent Foot Ulceration among Type 2 Diabetes Mellitus Patient: A Multi–Intervention Review. J. Public Health Res..

[39] Lira J.A.C., Nogueira L.T., Oliveira B.M.A., Soares D.R., Santos A.M.R., Araújo T.M.E. (2021). Fatores associados ao risco de pé diabético em pessoas com diabetes mellitus na Atenção Primária. Rev. Esc. Enferm. USP.

[40] Ghosh K., Dhillon P., Agrawal G. (2020). Prevalence and detecting spatial clustering of diabetes at the district level in India. J. Public Health.

